# In Vitro Biocompatibility Assessment of Nano-Hydroxyapatite

**DOI:** 10.3390/nano11051152

**Published:** 2021-04-28

**Authors:** Rafaela-Maria Kavasi, Catarina C. Coelho, Varvara Platania, Paulo A. Quadros, Maria Chatzinikolaidou

**Affiliations:** 1 Department of Materials Science and Technology, University of Crete, 70013 Heraklion, Greece; kavasiraf@gmail.com (R.-M.K.); vplatania@materials.uoc.gr (V.P.); 2 Foundation for Research and Technology Hellas (FORTH), Institute of Electronic Structure and Laser (IESL), 70013 Heraklion, Greece; 3 FLUIDINOVA, S.A., 4470-605 Maia, Portugal; catarina.coelho@fluidinova.pt (C.C.C.); paulo.quadros@fluidinova.com (P.A.Q.)

**Keywords:** hydroxyapatite, nanoparticles, cytotoxicity, genotoxicity, micronucleus test, cellular uptake, internalization, cosmetics and care products, toxicological and safety assessment

## Abstract

Hydroxyapatite (HA) is an important component of the bone mineral phase. It has been used in several applications, such as bone regenerative medicine, tooth implants, drug delivery and oral care cosmetics. In the present study, three different batches of a commercial nanohydroxyapatite (nHA) material were physicochemically-characterized and biologically-evaluated by means of cytotoxicity and genotoxicity using appropriate cell lines based on well-established guidelines (ISO10993-5 and OECD 487). The nHAs were characterized for their size and morphology by dynamic light scattering (DLS) and transmission electron microscopy (TEM) and were found to have a rod-like shape with an average length of approximately 20 to 40 nm. The nanoparticles were cytocompatible according to ISO 10993-5, and the in vitro micronucleus assay showed no genotoxicity to cells. Internalization by MC3T3-E1 cells was observed by TEM images, with nHA identified only in the cytoplasm and extracellular space. This result also validates the genotoxicity since nHA was not observed in the nucleus. The internalization of nHA by the cells did not seem to affect normal cell behavior, since the results showed good biocompatibility of these nHA nanoparticles. Therefore, this work is a relevant contribution for the safety assessment of this nHA material.

## 1. Introduction

Hydroxyapatite (HA, Ca_10_(PO_4_)_6_(OH)_2_) is a biocompatible ceramic material belonging to the calcium phosphates family. It is a widely used material in biomedical applications [1] and for bone and teeth tissue engineering due to its osteogenic properties to promote biomineralization [2,3]. The success of HA materials as bone implants is easily explained, since hydroxyapatite is the main component of the mineral phase of bones, comprising 65–70% of the human bone [1] and dentine and representing about 97% of the teeth enamel composition [4]. In bone, HA is present as nanocrystals that are aligned along collagen nanofibers. This nanostructure and its chemical composition are suitable to play a dual role, serving as hard material to provide mechanical support of the body and protection of important organs and maintaining calcium homeostasis. Moreover, the large specific surface area of HA nanocrystals favors the rapid dissolution by osteoclasts for maintenance of calcium ions in body fluids and appropriate bone remodeling rate [5]. Considering that HA is present in bone in nanosize in the shape of plates or needles of approximately 40 to 60 nm long, 5 to 20 nm wide, and 1.5 to 5 nm thick, the advances in nanotechnologies allowed the development of nanohydroxyapatite (nHA) materials to better mimic natural bone [6]. Several works evidenced that the inclusion of nHA improved the performance of several materials for bone regeneration in vitro and in vivo [7,8,9]. One of the most promising applications of nHA is in the treatment of osteoporosis, an important health hazard globally characterized by bone fragility. Bone mass is decreased, resulting in increased danger of fractures and decreased bone strength. In this situation, bone fractures could be filled with nHA-based medical devices [10]. More recently, nHA nanoparticles started to be used as an oral care ingredient in commercially available toothpastes, mouthwashes and other dental products [11,12]. Compared to other oral care ingredients, nHA showed superior performance in reducing dental hypersensitivity and improving enamel remineralization [11,13]. Moreover, nHA has been reported as a promising material for drug delivery applications, such as cancer treatment, rheumatoid arthritis and bone infections [14,15,16]. Although nHA nanoparticles have shown great performance in several applications, many questions have been raised regarding their safety. Particularly, in the last years, the Scientific Commission for Consumer Safety (SCCS) has been working to investigate the safety of nHA for oral care applications. At the moment, the SCCS does not have a final opinion regarding the safety of nHA for this application, since it is still necessary to clarify aspects such as genotoxicity [17,18]. Considering the aspects about the safety of nHA for its use in cosmetics and care products, this study is a relevant contribution to the toxicological assessment of this material.

Here, we report on the characterization and biocompatibility assessment of a commercial nanohydroxyapatite used in dental hygiene products. This nHA product was synthesized by wet chemical precipitation at room temperature in a continuous process described elsewhere [19]. The nHA samples were characterized to assess the nanoparticle morphology and size, as well as their chemical composition. For the in vitro cytotoxicity evaluation of the nHA samples, according to established guidelines, we assessed the cell viability and proliferation in direct contact with the nHAs. Moreover, we investigated their genotoxic potential by means of the micronucleus assay, and the cellular uptake of the nHAs was also visualized by transmission electron microscopy (TEM).

## 2. Materials and Methods

### 2.1. Hydroxyapatite Nanoparticles

A commercially available formulation of hydroxyapatite nanoparticles (nanoXIM·Care Paste, FLUIDINOVA, S.A., Maia, Portugal) was used, consisting of 15.5 ± 0.5% wt of hydroxyapatite nanoparticles dispersed in water. According to the manufacturer, this product has an average particle size <50 nm and potassium chloride content of 4.5 ± 0.5% wt. Three batches of hydroxyapatite designated as nHA-1, nHA-2 and nHA-3 were studied in this work to investigate eventual batch-to-batch variation. Depending on the assay performed, the nanoparticles were processed accordingly.

### 2.2. Physicochemical Characterization of the nHA Nanoparticles

#### 2.2.1. Transmission Electron Microscopy

TEM analysis was performed for the visualization of the three nanoparticulated samples nHA-1, -2 and -3. Dispersions of the nanoparticle samples were sonicated for 20 min, and a drop of each diluted sample was adjusted to a concentration of 75 μg/mL and placed on a carbon grid and allowed to dry overnight [20]. Next day, the samples were observed using a JEOL JEM-2100 (JEOL, Peabody, MA, USA) high-resolution transmission electron microscope. The size of the nanoparticles was measured from TEM images using the ImageJ software (National Institutes of Health, Bethesda, MD, USA). To determine the frequency distribution, a Gaussian (normal distribution) curve has been plotted [21] by means of the software GraphPad prism (GraphPad Software, San Diego, CA, USA) version 8.

#### 2.2.2. X-ray Diffraction (XRD)

XRD was used to evaluate hydroxyapatite phase composition. To perform this analysis, the hydroxyapatite nanoparticles suspension was previously dried overnight at 105 °C for water removal and then milled to obtain a fine powder. Diffractograms were obtained using a PANanalytical Empyrean (Malvern Panalytical, Malvern, UK) with Cu Kα radiation (λ = 1.5460 Å), voltages of 45 kV and 40 mA, an angle step of 0.0260° and 2θ range from 8 to 80°.

#### 2.2.3. Fourier Transformed Infrared Spectroscopy (FTIR)

For FTIR analysis, the hydroxyapatite suspension was dried and milled, as described for the XRD assay. The samples were analyzed as KBr pellets that were obtained by mixing 2 mg of each powdered samples with 200 mg of KBr. The mixture was placed in an uniaxial press (Graseby Specac, Orpington, UK) to obtain the discs. Infrared spectra were recorded using a PerkinElmer Frontier spectrometer (PerkinElmer, Waltham, MA, USA) with a resolution of 4 cm^−1^, 32 scans accumulated per sample and frequency regions from 400 to 4000 cm^−1^.

#### 2.2.4. Potassium and Chloride Quantification

The quantification of potassium in the hydroxyapatite samples was performed by atomic absorption spectrophotometry (AAS) with flame atomization using a Thermo 939 AA spectrometer (Thermo Fisher Scientific, MA, USA). The samples were previously dissolved in nitric acid and then analyzed using air/acetylene combustion gases at a flow rate of 0.8 mL/min and a hollow potassium cathode lamp at a wavelength of 769 nm in the working range of 0.5 to 4 mg/L of K^+^. For chloride quantification, ion chromatography (IC) was used by carrying out a bi-deionized water extraction with a Dionex Ionpac AS14A column. The analysis was performed in the working range of 2.0 to 30 mg/mL of Cl^−^ with a Dionex DX-120 equipment (Thermo Fisher Scientific, MA, USA).

#### 2.2.5. Dynamic Light Scattering (DLS)

Particles hydrodynamic radius of the three nHA samples was confirmed by dynamic light scattering (DLS) measurements in the very dilute regime of parts per million (after gradual dilutions of the initial nHA suspensions in deionized water) at three different angles, including 90° according to previous reports [22,23]. The measurements were carried out at 20 °C by means of an instrument ALV-5000 goniometer/correlator setup from the company ALV-Laser Vertriebsgesellschaft mbH Langen, Germany. The measurements for each sample were taken for 5 min per angle to have sufficient data points taken in order to get smooth correlation functions. The standard deviation of the diffusion coefficient is approximately 1.6% for this specific instrument, and the error of the analysis for the main process is approximately 5%.

### 2.3. Cell Culture Maintenance

The fibroblast-like cell line L929 (DSMZ Braunschweig, Germany, ACC-2) established from normal subcutaneous areolar and adipose tissue of a male C3H/An mouse was used for the cell viability and proliferation assessment as a relevant cell type for the biocompatibility testing of biomaterials according to the ISO 10993-5 standards. Cells were cultured in RPMI culture medium (biosera), supplemented with 10% Fetal Bovine Serum (FBS) 10% *v*/*v* (Gibco), 50 IU/mL penicillin (Sigma-Aldrich, St. Louis, MO, USA), 50 g/mL streptomycin (Sigma-Aldrich, St. Louis, MO, USA)] in a 5% CO_2_ incubator (Heal Force) at 37 °C.

The Chinese hamster (Cricetulus griseus) V79 cell line (lung fibroblasts) (DSMZ Braunschweig, Germany, ACC-335) was used for the genotoxicity assessment by means of the in vitro micronucleus method. This particular cell line is accepted by the OECD 487 guidelines for this assay. Cells were cultured in RPMI culture medium (biosera), supplemented with 10% Fetal Bovine Serum (FBS) 10% *v*/*v* (Gibco), 50 IU/mL penicillin (Sigma-Aldrich, St. Louis, MO, USA), 50 g/mL streptomycin (Sigma-Aldrich, St. Louis, MO, USA) in a 5% CO_2_ incubator (Heal Force) at 37 °C.

### 2.4. Cell Cytotoxicity Assessment by Cell Viability Evaluation

Cell viability was evaluated according to ISO 10993-5 (2009) standards and measured by the PrestoBlue^®^ viability assay (Invitrogen) according to a protocol based on the manufacturer instructions [24]. For the assessment of the nHA samples, 10^4^ cells per well were seeded in 96 well plates on day 0 and on day 1 culture medium was replaced with medium containing the nHA samples. Cell viability was measured at days 1 and 2. The measurements were performed in a spectrophotometer (Synergy HTX Multi-Mode Microplate Reader, BioTek, Bad Friedrichshall, Germany) and the absorbance was measured at 570 and 600 nm. The term control refers to cells cultured on the tissue culture treated polystyrene (TCPS) surface. Data represent means ± standard deviation of quadruples of three independent experiments (n = 12).

### 2.5. Genotoxicity Assessment According to OECD 487 (Micronucleus Assay)

For the genotoxicity assessment, 3 × 10^4^ of the V79 cells per well were seeded in 48 well plates, followed by 24 h incubation at 37 °C with 5% CO_2_. Methyl methanesulfonate (MMS) was used as positive control, as suggested by OECD 487 guidelines, and cells seeded on TCPS were used as negative control. The concentrations of the nHAs used were 0.5, 1, and 2 mg/mL, and this is in line with the OECD 487 guidelines (2019) based on the statement described in page 7: “If no precipitate or limiting cytotoxicity is observed, the highest test concentration should correspond to 10 mM, 2 mg/mL or 2 μL/mL, whichever is the lowest”. Next, fresh culture medium was added with the nano-HA samples (0.1% *v*/*v*) in 300 μL total volume per well. After 24 h incubation, the fixation and staining were performed as follows: culture medium was removed, and the samples were washed twice with PBS. Fixation was achieved with 4% *w*/*v* paraformaldehyde (PFA) for 20 min. Then, after two washes with PBS, staining of the DNA was performed with a Giemsa solution (Giemsa 3% in Na_2_HPO_4_ and K_2_HPO_4_ 0.6 M each, pH 6.8) for 40 min and washed twice with PBS. Finally, the number of micronuclei was counted under an optical microscope (2000–3000 cells per measurement). Data represent means ± standard deviation of triplicates of three independent experiments (n = 9).

### 2.6. Visualization of Nanoparticle Uptake with TEM

TEM analysis was performed for the visualization of the nHA samples uptake by pre-osteoblastic cells, as a proved cellular system for nanoparticles uptake by cells at a concentration of 0.25% *v*/*v* by means of a high resolution transmission electron microscope, according to the methodology described earlier [25]. Briefly, the cells incubated for 24 h with the three nHA samples (and the control cells without samples) were detached from the culture flask, washed with phosphate buffer saline (PBS), and fixed in 2% (*v*/*v*) glutaraldehyde, 2% (*v*/*v*) paraformaldehyde in 0.1 M sodium cacodylate buffer (SCB), pH 7.4, for 24 h at 4 °C. The samples were washed three times for 5 min each in 0.1 M SCB, postfixed in 1% aqueous OsO_4_ for 12 h at room temperature, and then washed again. After the last washing step, the samples were stained with 2% (*v*/*v*) uranyl acetate for 1 h. Then, the samples were rinsed with SCB, dehydrated through an ascending acetone gradient of 30, 50, 70, 90, 100% (*v*/*v*), infiltrated with Durcupan ACM Fluka resin [3:1 propylene oxide: resin mixture for 1 h followed by a 1:1 and a 1:3 propylene oxide:resin mixture for 1 h each and finally 100% (*v*/*v*) resin for 16 h], and embedded in flat molds. The resin was cured in a drying oven at 60 °C for 48 h. The samples were trimmed, thin-sectioned, and absorbed onto 300-mesh copper grids. Observation was carried out using a high-resolution transmission electron microscope JEOL JEM-2100HR (JEOL, Tokyo, Japan) at an operating voltage of 80 kV.

### 2.7. Statistical Analysis

Statistical analysis was performed for the cell viability and genotoxicity assessment with micronucleus formation using the one-way ANOVA Dunnett’s multi-comparison test in GraphPad Prism version 8 software (GraphPad Software, San Diego, CA, USA). *p*-Values indicate statistically significant differences.

## 3. Results

### 3.1. Physicochemical Characterization

#### 3.1.1. TEM Analysis

**Figure 1:**
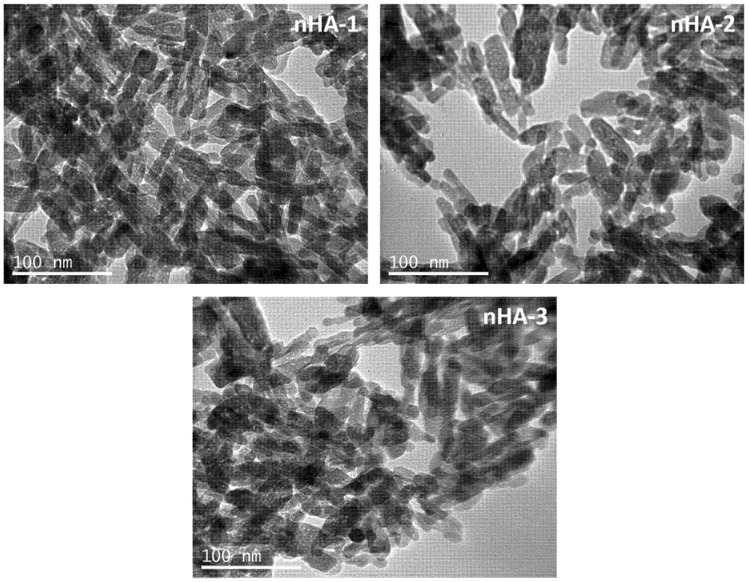
Transmission electron microscopy images from nHA-1, -2 and -3 indicating a rod-like shape of an average length of approximately 20 to 40 nm. The scale bar represents 100 nm.

**Figure 2:**
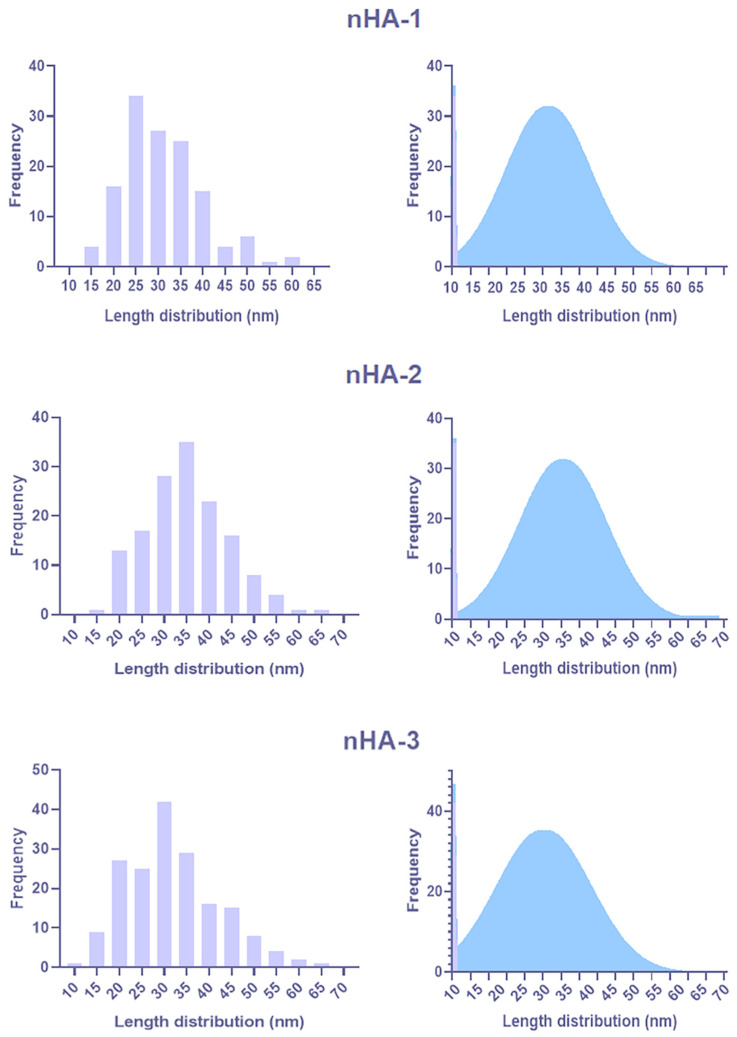
Particle size distribution determined using image analysis of TEM images. The size distribution is presented as histograms (**left**) and the correspondent Gaussian curves (**right**).

TEM images from the three nHA batches are presented in Figure 1 and show the size and structure of nHA-1, -2 and -3. No significant differences of size or morphology were observed between the 3 batches analyzed. Representative TEM images depict that all three nHA-1, -2 and -3 materials have a rod-like shape and tend to form agglomerates. The particle size distribution shows that particles have an average length of approximately between 20 and 40 nm (Figure 2).

#### 3.1.2. XRD

**Figure 3:**
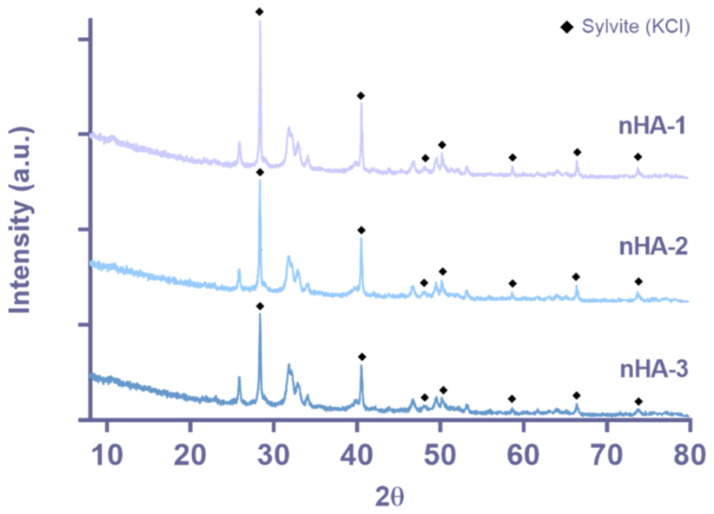
XRD diffractograms for nHA-1, -2 and -3.

HA phase composition was assessed by XRD, and the obtained diffractograms can be observed in Figure 3. The presence of sylvite (KCl) was detected for the three samples. This result was expected, since these nHA suspensions have a small percentage of KCl, as referred by the manufacturer, and also confirmed in this work by atomic absorption spectrophotometry (AAS) and ion chromatography (IC). Besides the KCl, the peaks at approximately 26° and 32° (2θ) present in the diffractograms correspond to the hydroxyapatite phase [2]. The intensity of the [002] peak at approximately 26° is almost comparable with the intensity of the characteristic peak at 32° pointing on the preferred orientation of the nanocrystals in the elongated z-direction. Moreover, the width of the [002] peak is sharper than any other HA peak, which confirms—based on the Scherrer equation—that the crystals are larger in z-direction compared to all other directions, and this is in agreement with the elongated structure of the nHA particles visible from the TEM images. Moreover, the average crystallite size was determined, and the values of 26.7 ± 1, 24.9 ± 5 and 26.2 ± 5 nm were obtained for sample nHA-1, nHA-2 and nHA-3, respectively. The patterns of the three diffractograms present no relevant differences, and therefore it can be concluded that there are no differences between the three batches in terms of phase composition and rod-like shape of the nanoparticles.

#### 3.1.3. FTIR

**Figure 4:**
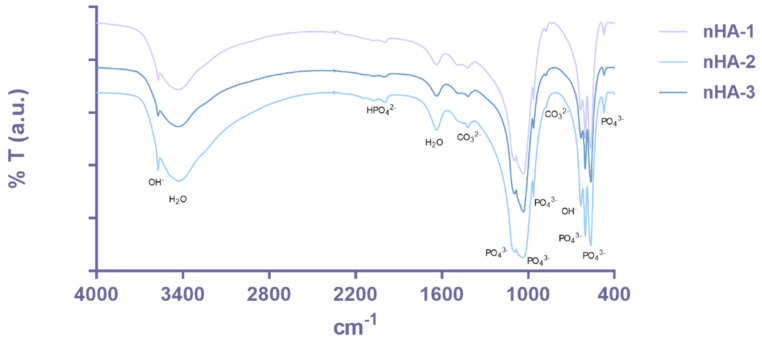
FTIR spectra for nHA-1, -2 and -3 samples.

The chemical composition of the samples was also investigated using FTIR to evaluate the presence of functional groups at the molecular level (Figure 4). The spectra obtained for the three samples showed the hydroxyapatite characteristic peaks, particularly the phosphate groups at 473, 564, 603, 962, 1032 and 1094 cm^−1^ [26,27]. These phosphate group peaks can be related with an average particle size. The dependence of vibrational frequencies on the size of phosphate groups could be analyzed in the mid- and far-IR domain, as described elsewhere [28], however, this was not subject of this work. Moreover, the OH^−^ vibrational and stretching modes were also evident at 633 and 3572 cm^−1^. As expected, lattice water was also present in the samples and represented by the peak at 1638 cm^−1^ and the broad band 3200–3500 cm^−1^ [26,27]. Peaks at 877 and 1421 cm^−1^ were related with carbonate groups in the samples that can result from the CO_2_ adsorption by apatite [29]. All samples also present weak peaks at 2002 and 2076 cm^−1^ that might correspond to HPO_4_^2−^ as previously reported in other studies [29,30]. It is possible to relate the dependence of phosphate groups as a function of average size.

#### 3.1.4. AAS and IC

AAS and IC were used to quantify the concentration of potassium and chloride present in the samples. An average concentration of 2.1 ± 0.1% and 2.3 ± 0.1% were obtained for potassium and chloride, respectively (Table 1). These results confirm the product specification of 4.5 ± 0.5% wt indicated by the manufacturer.

#### 3.1.5. DLS

**Figure 5:**
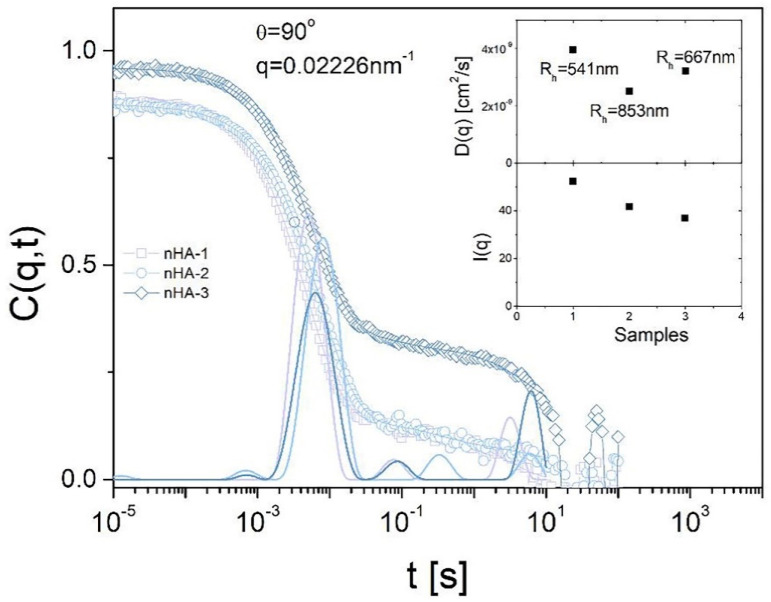
Dynamic light scattering analysis of the three samples nHA-1, -2 and -3 to determine particles hydrodynamic radius R_h_ of the three nHA samples measured in high dilutions of parts per million in deionized water at 90° angle. The standard deviation of the diffusion coefficient is approximately 1.6% and the error of the analysis for the main process is approximately 5%.

Particles hydrodynamic radius R_h_ of the three nHA samples was confirmed by DLS measurements in the dilute regime of parts per million (after gradual dilutions of the initial nHA suspensions) at an angle of 90° (for all nHAs) and three different angles for nHA-1. The DLS graph in Figure 5 shows a comparison of the three nHAs at the measurement angle of 90°. We assume that the distribution of the relaxation times (thick solid curves, main graph) is translational diffusion. Since we measured in dilute condition, it is safe to assume that the main mode follows translational diffusion (Brownian motion). The graph of the upper inset indicates an apparent hydrodynamic radius, which is related to the actual size, shape and particle-solvent interactions. From the correlation function (open symbols main plot) and its CONTIN analysis (fit and distribution), we conclude that in the 3 different nHA samples, there is mainly one big species and some very large agglomerates (that are low in concentration). For nHA-2 and nHA-3, most of the particles appear with apparent sizes around 500–850 nm, and a few larger agglomerates are present. Although data were not taken from many different angles in DLS for nHA-2 and nHA-3, which would lead to a conclusion that size distribution of the main mode would be polydisperse, it is very likely that they are polydisperse given that the main mode is an agglomerate of cylinders/rods.

### 3.2. Cytotoxicity Assessment

**Figure 6:**
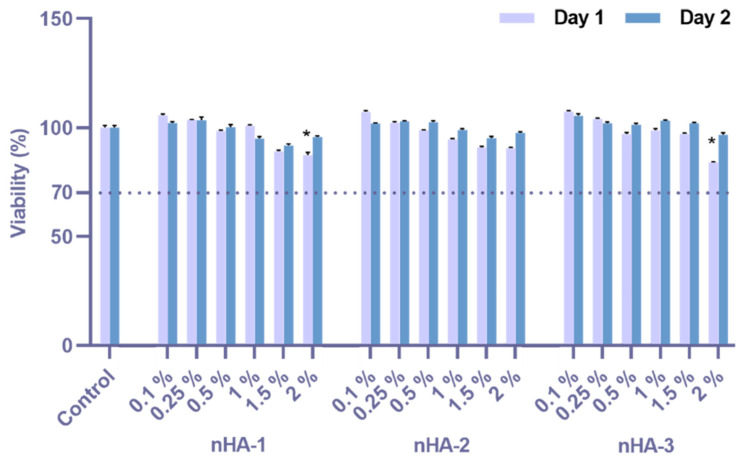
Assessment of cytotoxicity of nHA-1, -2 and -3 samples at the concentrations of 2% (corresponds to 3.1 mg/mL), 1.5%, 1%, 0.5%, 0.25%, and 0.1% *v*/*v* according to ISO 10993-5 (2009) using the resazurin-based cell viability reagent PrestoBlue^®^. Cell viability of L-929 fibroblasts was expressed as % of the control (control was set at 100%). The values represent means ± standard deviation of quadruples of three independent experiments (n = 12), and the asterisk (*) designates significant differences compared to the TCPS control (*p* = 0.0001).

The cell viability was quantified using six different concentrations (0.1, 0.25, 0.5, 1.0, 1.5 and 2% *v*/*v*) of the three nHA samples using L929 murine fibroblasts after 1 and 2 days in culture in direct contact with the materials, and the % viability to the TCPS control (cells only, set at 100%) are graphically presented (Figure 6). All three nHA samples present a cell viability of 90 to 100% compared to the control on both time points (24 and 48 h) at all six investigated concentrations. For nHA-1 and nHA-3, we observed at the highest concentration of 2% *v*/*v* a cell viability of 87% and 84%, respectively. According to the ISO 10993-5, a limit of 70% cell viability considers the samples cytocompatible. All the samples investigated are above 70%, therefore highly biocompatible. Statistical analysis indicates significant differences (*p* = 0.0001) for nHA-1 and nHA-3 at the concentration of 2% *v*/*v* compared to the TCPS control.

### 3.3. Genotoxicity Assessment by Means of the Micronuclei Formation

**Figure 7:**
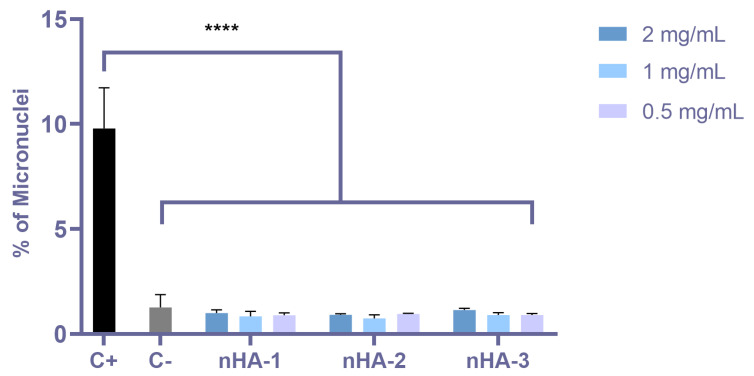
Genotoxicity assessment of the three nHA samples according to the OECD 487 in V79 cells. The graph shows the % of micronuclei formation in stained V79 cells. The concentrations of the three nHAs used were 0.5, 1, and 2 mg/mL. Positive control (C+): 400 μM methyl methane sulphonate (MMS); negative control (C−): cells only. Micronuclei were measured in 2000–3000 cells in each experiment. The values represent means ± standard deviation of triplicates of three independent experiments (n = 9). The asterisk (****) designates significant differences of all three nHAs and the TCPS control (C−) compared to the MMS positive control (C+) (*p* < 0.0001).

Micronuclei formation was measured upon 24 h incubation of V79 cells with the nHA samples, according to the OECD 487 standards. The results are presented graphically as per cent micronuclei of the cells (Figure 7). The three nHAs present a very low percentage of micronuclei, lower or similar to the negative control (cells only), indicating that they are not genotoxic.

### 3.4. Uptake of nHA Samples by Cells Investigated by TEM

**Figure 8:**
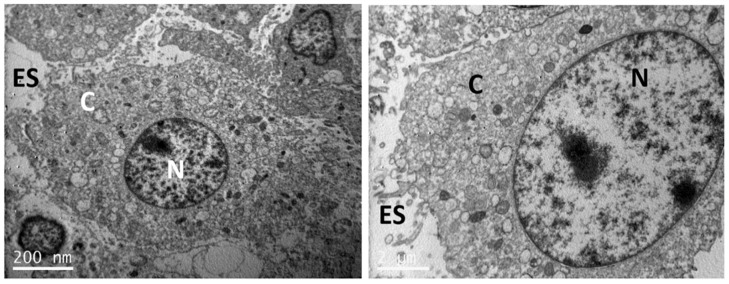
Representative TEM images showing pre-osteoblastic cells (control) in the absence of any nHA samples. The scale bar represents 200 nm (**left**) and 2 μm (**right**). The letter N designates the nucleus, C the cytoplasm and ES the extracellular space.

**Figure 9:**
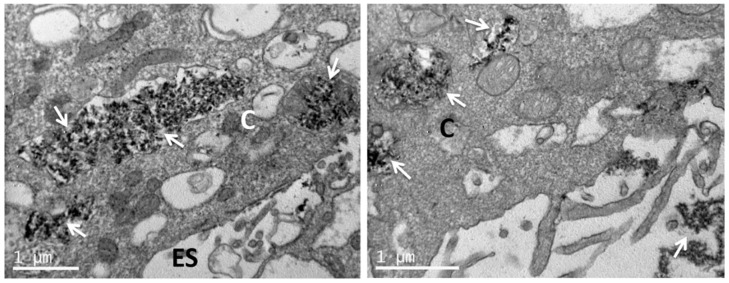
Representative TEM images showing nHA-1 uptaken by pre-osteoblastic cells. Nanoparticles of nHA-1 are shown localized and presented in both the extracellular and the intracellular space in cyst-like structures. The scale bar represents 1 μm. The letter C designates the cytoplasm and ES the extracellular space. The arrows depict the nanoparticulated sample nHA-1.

**Figure 10:**
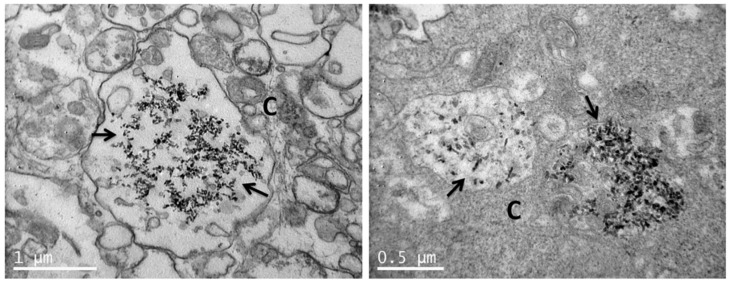
Representative TEM images showing nHA-2 uptaken by pre-osteoblastic cells. Nanoparticles of nHA-2 are shown localized in the intracellular space in vesicles (in other images that are not shown, they appear also in the extracellular space). The scale bar represents 1 μm (**left**) and 0.5 μm (**right**). The letter C designates the cytoplasm, and the arrows depict the nanoparticulated sample nHA-2.

**Figure 11:**
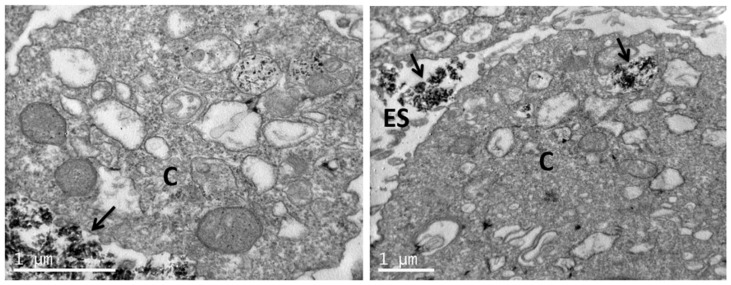
Representative TEM images showing nHA-3 uptaken by pre-osteoblastic cells. Nanoparticles of nHA-3 are shown localized in both the extracellular and the intracellular space in cyst-like structures. The scale bar represents 1 μm. The letter C designates the cytoplasm, ES the extracellular space, and the arrows depict the nanoparticulated sample nHA-3.

TEM images of sections of pre-osteoblastic cells in the absence of any nHA material (control) are depicted in Figure 8, whereas the uptake of the nHA-1, -2 and -3 samples by the cells is visualized in Figure 9, Figure 10 and Figure 11, respectively. TEM analysis reveals that all three samples can be uptaken by pre-osteoblastic cells. In representative TEM images, nanoparticles of all three nHA types nHA-1, -2 and -3 are shown localized in both the extracellular space outside and between cell membranes, and also in the intracellular space in cytoplasm within cyst-like structures. For all the samples, the nHA product was not observed in the cell nuclei since the nHA morphology was not identified inside the nuclei.

## 4. Discussion

The main goal of this study was to assess the biocompatibility of a commercially available nHA product used in bone regenerative applications and as an oral care ingredient. Three nHA samples were used to show the reproducibility of this product and evaluate possible differences between batches. The nanoparticle characterization assays showed that the three batches have similar physicochemical characteristics. Particularly, all samples show nano-rod shaped nHA particles with a length between 20 and 40 nm, and a tendency to form big agglomerates, as evidenced by DLS and TEM. Moreover, chemical analysis showed the presence of HA and KCl, with KCl being present in a concentration below 5% for all samples. The characterization assays confirmed the reproducibility of this nHA product, since all samples showed similar results. The three nHA samples were investigated in cell culture in several concentrations ranging from 0.1 to 2% *v/v*, in order to be able to make a conclusion regarding the safety of their use at the in vitro level. The results of the viability assay were satisfactory, as no significant changes were observed, compared to the TCPS control. Importantly, all three nHA types revealed similar results, showing a good reproducibility in the manufacturing of the samples. This is in agreement with a previous study on cytotoxicity assessment of similar nHA materials [18,26]. It has been shown that some HA nanoparticles could be cytotoxic depending on their characteristics, such as the manufacturing procedure [31], but the samples of this study did not present cytotoxicity at all, implying a successful way of production. Furthermore, the significance of the good cytotoxicity results of this nHA material is justified by the fact that other HA samples were found toxic and inhibited cell proliferation of human hepatoma cells [32].

Apart from cytotoxicity, genotoxicity is an equally important factor when a material is made for human use. The DNA damage is not easily detectable, and the individual could occur problems upon long-term use of a genotoxic product. Based on the OECD 487 guidelines, the genotoxicity was assessed by means of the micronuclei formation method. V79 cells were chosen for this method without the addition of cytochalasin, according to OECD 487. All three nHAs presented similar micronuclei percentage to the TCPS control, while the MMS presented significantly higher micronuclei number. In another report, genotoxicity assessment of hydroxyapatite/bioactive glasses did not reveal DNA damage [33]. Interestingly, an in vivo study on rats demonstrates non-genotoxic effects of HA. Genotoxicity was investigated in blood, liver, kidney and lung after 30 days of HA implantation, and the results were satisfying for both cytotoxicity and genotoxicity, showing the safety of HA in tissue engineering [34]. In another tissue engineering application, in which HA was combined with silk fibroin membranes, neither cytotoxicity nor genotoxicity were reported [35].

Nanoparticles uptake by the cells was also evident as the nanoparticles were visualized into the cytoplasm. Another positive observation is that the nanoparticles do not enter the nucleus of the cell. This observation also supports the results of lack of genotoxicity. Similarly, in another study, the cellular uptake of larger HA nanoparticles has been observed. In agreement with the present study, the nanoparticles were internalized and captured inside vesicles into the cytoplasm [36]. Importantly, HA nanoparticles have been previously shown to be internalized by MC3T3-E1 pre-osteoblastic cells [37], while calcium phosphate nanoparticles carrying BMP-7 plasmid elicited an increase in the osteogenic response in the same cell type upon successful uptake and transfection [38]. Furthermore, HA nanoparticle uptake was also demonstrated in breast cancer, with an internalization mechanism based on syndecan-4 [39].

## 5. Conclusions

The commercial nHA product investigated in this work showed good reproducibility, since the three batches tested (nHA-1, -2 and -3) presented similar physicochemical properties. Particularly, all three nHA samples tend to form big agglomerates, as visualized by means of TEM. Moreover, TEM images provided information about the nanoparticle size of the samples, indicating that all three nHAs have a rod-like shape with an average length of approximately 20 to 40 nm. All the three samples (nHA-1, -2 and -3) presented high cell viability, similar to the TCPS control, in both experimental time points of 24 and 48 h in culture at all six concentrations investigated from 0.1% *v*/*v* to 2% *v/v,* revealing no cytotoxic effects. Importantly, a low number of micronuclei was observed in the genotoxicity assessment, hence mutagenicity is not observed. TEM analysis was also used to reveal the nanoparticle uptake by osteoblastic cells, as the HA nanoparticles were observed into the cytoplasm. The nHA was not observed in the nucleus, and this result is in agreement with the genotoxic evaluation. Considering the aspects about the safety of nHA required by the SCCS for its use in cosmetics, this study is a relevant contribution to the toxicological assessment of this nHA material, since its excellent cytocompatibility and absence of genotoxicity are clearly demonstrated, rendering it suitable for commercial use.

## Figures and Tables

**Table 1:** Potassium and chloride quantification for the samples nHA-1, -2 and -3.

	K^+^ (%)	Cl^−^ (%)
**nHA-1**	2.2	2.3
**nHA-2**	2.0	2.3
**nHA-3**	2.0	2.2
**Average**	2.1	2.3
**SD**	0.1	0.1

## Data Availability

Data supporting reported results will be provided by the authors upon request.
